# Neuroscience Networks: Data-sharing in an Information Age

**DOI:** 10.1371/journal.pbio.0000017

**Published:** 2003-10-13

**Authors:** Thomas R Insel, Nora D Volkow, Ting-Kai Li, James F Battey, Story C Landis

## Abstract

To study the brain from molecules to behaviour, neuroscientists face the challenge of communicating an emerging wealth of information in coherent accessible forms

The completion of the human genome project has ushered in a new era in which biology has become an information science. In this new era, sharing of information is quickly becoming a critical aspect of scientific discovery. As directors of National Institutes of Health (NIH) institutes dedicated to neuroscience, we recognize several areas of research where sharing of primary data will be necessary for us to reach our scientific goals, including brain-mapping, genetics, and clinical trials. Progress in each of these areas will require not only new tools for sharing information but a change in our scientific culture. Here we describe some of the recent progress in efforts to map the brain as an example of the potential and the challenge of sharing data in an era when neurobiology, like genomics, is becoming an information science.

In parallel to the worldwide effort to map the human genome, investigators in neuroscience have used a range of techniques to map the brain. The efforts share some superficial similarities: the genome has 3 × 10^9^ bases and the human brain has roughly 100 × 10^9^ neurons; both the genome and the brain have embedded modules of functional units (genes versus circuits) that can be mapped in space; and localization of both genes and circuits requires computational power that can be distributed across laboratories. But the analogy breaks down quickly. Whereas fundamental genome data can be addressed as unidimensional text of four letters in varying order, a comprehensive map of the brain includes molecular, cellular, system, and behavioral data—all of which are dynamic, interacting, and interdependent. For example, brain circuitry is organized in three-dimensional space constantly changing in time, with each neuron having 10^3^–10^4^ synapses and with many of those synapses capable of plasticity that may, in turn, have significant functional consequences.

As we emerge from the “decade of the brain,” we are entering a decade for which data-sharing will be the currency for progress in neuroscience.

As a testament to the complexity of brain data, a century after the classic age of neurohistology, there are continuing arguments about the taxonomy of neurons, depending on location, morphology, neurochemistry, or RNA profile. For instance, a population of neurons in a small region of the brain, the dorsal raphe, is the main source of the neurochemical serotonin that has been implicated in stress responsiveness and mood disorders. These serotonergic neurons can be subdivided according to rostralcaudal location, axon thickness, or projections (Mamounas et al. 1991; Lowry 2002). However, what we recognize by immunochemical stain as a single shared phenotype in an anatomically distinct region may consist of a heterogeneous population of cells with diverse RNA profiles. In this sense, the strategy for brain-mapping might borrow a page from astronomy, with its maps of galaxies with mixed elements, as well as from the experience of the genome project.

Indeed, advances in human brain-mapping, like discoveries in astronomy, have until recently largely depended on the tools available. The postmortem studies of the early 20th century provided delineation of cortical areas through light microscopic histology and gross connectional information. Neurochemical techniques in the last three decades yielded maps with cellular and subcellular resolution, identifying populations of cells usually by one or two neurochemical phenotypes. During the same period, electrophysiological approaches revealed the exquisite distribution of function across the brain, within particular brain subdivisions, and within neurons themselves. In the past two decades, direct study of the intact, functioning human brain in healthy and disordered states has been made possible by a variety of neuroimaging modalities. These studies have provided both structural and functional topography at increasing resolution, as well as neurochemical data and, most recently, information regarding neural connectivity (Behrens et al. 2003). The advent of techniques for mapping RNA profiles now permits analysis of several thousand species of RNA even in a single neuron, resulting in exponential increases in information. As these approaches are combined with the experimental behavioral and clinical sciences, opportunities abound for understanding this complex organ and treating its pathologies.

The challenge now is to integrate this information into a coherent, accessible form that permits hierarchical analysis from RNA to protein to morphology to connectivity to function in a universal language while preserving fidelity. While earlier comprehensive maps in simpler nervous systems, such as the classic lineage maps of invertebrates (Stern and Fraser 2001), could be completed by single labs, more ambitious projects like a transcriptional map of the mouse brain, the Human Genome Project, and other goal-directed or large-scale research endeavors (Nass and Stillman 2003) will require *collaboration* of scientists who add value to the enterprise by working in multidisciplinary teams; *coordination* of efforts to attain a goal; and *computation* through the use of informatics, models, and simulations. The keystone in this new paradigm is, of course, meaningful data-sharing.

Several initiatives serving the brain and behavioral research communities are advancing cooperative research. The Gensat Project (www.gensat.org) will soon provide developmental and whole-brain maps of several hundred genes in the mouse nervous system using a bacterial artificial chromosome (BAC) transgenic strategy with fluorescent reporters to provide subcellular resolution. A digital atlas of the mouse brain and associated informatics tools have been developed to organize, visualize, and analyze such gene expression (and other spatial) data generated by researchers (http://www.loni.ucla.edu/MAP/index.html). We now have the capability to map the transcriptional expression of virtually the entire mouse genome in the adult and the developing mouse brain, registering these data to a common, digital atlas. Like the galaxy maps generated by the Hubble telescope, this transcriptional atlas will provide important temporal as well as spatial information, revealing genes that may be expressed only at critical stages of brain development. Similarly, the Human Brain Project (http://www.nimh.nih.gov/neuroinformatics/index.cfm) is an informatics effort funded through several federal agencies to develop databases, analytical and computational simulations, and other resources to assist human brain-mapping as well as other large-scale coordinated neuroscience programs.

While there are several initiatives at NIH aimed at overcoming the informatics barriers to sharing data and facilitating collaboration, coordination, and computation, we recognize that not all of the impediments to data-sharing are technical. The advent of neurobiology as an information science also demonstrates that the academic culture in which our science develops and the publication culture in which our science is communicated will need to change. Promotion decisions at major universities largely depend on the quality and quantity of first-authored or senior-authored papers. Multidisciplinary studies require teams of investigators in which hierarchical schemes for authorship may fail to reflect accurately the magnitude of each individual's contributions. Similarly, contributions to a research database may represent important scientific and scholarly achievements, but generally are underrecognized by promotions committees counting peer-reviewed publications. Indeed, the nature of publication itself needs to change in an era when some of the most important contributions will emerge from comprehensive descriptions of new landscapes (analogous to new genomes and new galaxies) rather than tests of specific hypotheses. These cultural issues are not peculiar to brain and behavioral science, of course, and have recently been considered broadly at the NIH (http://www.becon.nih.gov/symposium2003.htm).

Scientific publication, as we have known it in print, is slow and expensive, with access limited to those with either the funds to purchase an individual subscription or the proximity to a library with an institutional subscription. Data-sharing also means open-access publishing so that data, whether from mapping efforts or from hypothesis-driven experiments, become available quickly and freely to the scientific community. As we emerge from the “decade of the brain,” we are entering a decade for which data-sharing will be the currency for progress in neuroscience. Efforts driven by collaboration, coordination, and computation should yield the data, tools, and resources that neuroscientists will need in the coming decades. We hope that new electronic publications with open access will accelerate this change and provide the vehicle for disseminating the most exciting discoveries in neuroscience in a rapid, respected, and ready format. 

**Figure:**
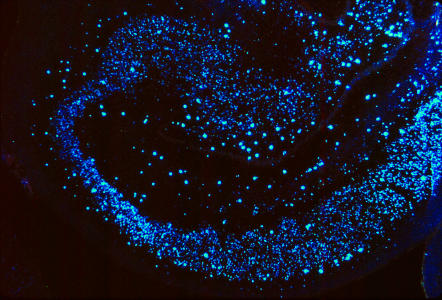
A Constellation of Neurons Image courtesy of Miles Herkenham.
